# Ethanolic Extract of *Vitis thunbergii* Exhibits Lipid Lowering Properties via Modulation of the AMPK-ACC Pathway in Hypercholesterolemic Rabbits

**DOI:** 10.1155/2012/436786

**Published:** 2012-03-28

**Authors:** Chun-Hsu Pan, Chia-Hua Tsai, Wen-Hsin Lin, Guo-Yan Chen, Chieh-Hsi Wu

**Affiliations:** ^1^School of Pharmacy, China Medical University, Taichung 40402, Taiwan; ^2^Research and Development Department, Medigreen Biotechnology Corporation, Taipei 22046, Taiwan

## Abstract

*Vitis thunbergii* (VT) is a wild grape that has been shown to provide various cardioprotective effects. The present study was designed to examine whether a VT extract could reduce serum lipid levels and prevent atherogenesis in a hypercholesterolemic rabbit model. At the end of an 8-week study, our results showed that a VT extract supplement markedly suppressed the serum levels of cholesterol and low-density lipoprotein, reduced lipid accumulation in liver tissues, and limited aortic fatty streaks. Our findings suggest that the VT extract activated AMPK (5′-adenosine monophosphate-activated protein kinase) with subsequent inhibition of the activation of ACC (acetyl-CoA carboxylase). Our results suggest that this VT extract could be further developed as a potential lipid-lowering agent and as a natural health food to prevent atherogenesis.

## 1. Introduction

The treatment and prevention of cardiovascular diseases, particularly atherosclerosis, remains a critical research topic for modern medicine. Atherosclerosis is a slow progressive disease and is the most common cardiovascular disease among Western societies, where it remains the leading cause of both illness and death. Atherosclerosis may be initially caused by damage to the inner layers (endothelium) of the arteries from several risk factors, which include hypercholesterolemia (or hyperlipidemia), hypertension, diabetes, and cigarette smoking. Lipid-lowering agents, which include statins and fibrates, reduce the blood levels of fats such as cholesterol and triglycerides and have become some of the most common and effective prescribed drugs for the treatment of atherosclerosis. Nevertheless, the clinically adverse side effect of myotoxicity has been associated with the use of lipid-lowering drugs, and this condition may eventually lead to renal failure and death in the worst cases [1]. Accordingly, developing new lipid-lowering agents or natural supplements is an important issue that warrants further study.

AMPK (5′-adenosine monophosphate-activated protein kinase) is a known physiological cellular senor for energy homeostasis. This enzyme is composed of three parts (*α*, *β*, and *γ* subunits) that bind together to make a functional kinase. The energy-sensing capability of AMPK can be attributed to its ability to detect and react to the changes in the ratio of AMP/ATP molecules [2]. Under conditions of fasting or an insufficient energy supply, activated AMPK regulates several intracellular metabolic systems to either generate energy or reduce energy depletion. These metabolic changes include increases in the cellular uptake of glucose through inhibiting gluconeogenesis [3, 4] and the upregulation of the glucose transporter type 4 (GLUT4) [4, 5], the acceleration of lipid catabolism *via* the suppression of acetyl-CoA carboxylase (ACC) [6], inhibition of cholesterol synthesis via depressed activity of HMG-CoA reductase (HMGCR) [7], and decreased fatty acid *de novo* biosynthesis via suppression of fatty acid synthase (FAS) [8]. Accordingly, AMPK protein has the potential to be a therapeutic drug target for the treatment of hyperlipidemia or atherosclerosis.

*Vitis thunbergii *(VT) is a wild grape that is native to Taiwan and East Asia. It is widely used in medicinal wines and beverages. Several natural components isolated from *V. thunbergii* have been shown to provide various beneficial pharmacological effects, including antiinflammation [9, 10], antioxidation [11], antitumor [12], antiplatelet aggregation [11], antimicrobial [13], and neuroprotective activities [14, 15]. Although some of the isolated compounds from *V. thunbergii* have cardioprotective effects, their effects on lipid levels and atherosclerosis are unknown. Therefore, the purpose of the present study was to evaluate whether *V. thunbergii* could reduce serum lipid levels and prevent atherosclerosis formation. This study also investigated whether the molecular mechanisms underlying the lipid-lowering effects of *V. thunbergii* are associated with AMPK pathway activation.

## 2. Materials and Methods

### 2.1. Materials

*V. thunbergii* was kindly provided and plant origin identified by Dr. Tzer-Kaun Hu (Department of Agronomy, National Chung Hsing University, Taiwan). Anti-phospho-ACC (#3661) and anti-phospho-AMPK*α* (#2531s) were obtained from Cell signaling Technology, Inc. (Beverly, MA, USA). The primary antibodies against *β*-actin (#ab6276) and HMGCR (#07-457) were purchased from Abcam (Cambridge, MA, USA) and Millipore/Upstate (Bedford, MA, USA), respectively. Horseradish peroxidase-labeled secondary antibodies against mouse IgG (#sc2005) and rabbit IgG (#sc2004) were purchased from Santa Cruz Biotechnology (Santa Cruz, CA, USA). All other reagents were purchased from Sigma-Aldrich (Louis, MO, USA).

### 2.2. Preparation of *V. thunbergii* Extract

*V. thunbergii* was homogenized to a powder. Subsequently, the homogenized material (about 1 kg) was soaked in 10 L of 50% ethanol solution (extractive solvent) at 80°C for 1 hr. The solid residue of the extracted herbs was filtered and discarded through a Buchner funnel lined with Whatman filter paper. The filtrate was concentrated to a paste by distillation under reduced pressure. The concentrated *V. thunbergii* extract was further diluted with deionized water for all of the subsequent experiments.

### 2.3. Experimental Model

Twenty-four male New Zealand White rabbits (average of six-week-old) were purchased from Lu-Hop ranch (Changhua, Taiwan). The animals were housed in individual cages with free access to water and maintained on a 12 hr light/dark photocycle. All animal care followed the institutional animal ethical guidelines of China Medical University. After 1 week of adaptation, the rabbits were randomly divided into four groups, which were fed the following daily treatment diets for 8 weeks: a regular diet (Control group; FwuSow Ind., Taiwan), 0.5% (w/w) cholesterol diet alone (CHOL group), 0.5% (w/w) cholesterol diet with 0.01% (w/w) lovastatin supplement (LOVA group; YungShin Pharm. Ind., Taiwan), and 0.5% (w/w) cholesterol diet with a 7% (w/w) *V. thunbergii* extract supplement (VT group). The daily feeding amount for each rabbit was 50 g/kg body weight per day. At the beginning and end of the 8-week study, the rabbits were anesthetized by an intramuscular injection of Zoletil 50 (1 mL/kg) (Virbac Ltd., France), and blood samples were harvested. Finally, the aortas (from aortic arch to the bifurcation of the iliac arteries) and whole livers were collected from the rabbits after they were sacrificed for further histopathological and western blot analyses.

### 2.4. Blood Chemistry Analysis

The animals were fasted overnight before blood drawing. The blood was collected from the marginal ear veins of rabbits into BD Vacutainer EDTA Blood Collection Tubes. Plasma was separated by centrifugation at 3,000 rpm at 4°C for 10 min. Measurements for changes in blood chemistry parameters included serum levels of low-density lipoprotein (LDL), cholesterol (Chol), triglycerides (TG), glutamate oxaloacetate transaminase (GOT), and glutamate pyruvate transaminase (GPT) (ZhenXing Co., Ltd, Taiwan).

### 2.5. Cryosectioning of Liver Tissues

The rabbit liver tissues were perfused with normal saline and fixed in 10% (v/v) formalin-neutralized solution (J.T. Baker, Inc., USA) for 24 hr. Afterward, the tissues were embedded in Tissue-Tek OCT Compound (#4583; Sakura Finetek Inc., USA). Embedded tissues were cut into 10 *μ*m thick slices and stained with Sudan IV and hematoxylin (Merck, USA). Briefly, the slices were washed with pure water for 1 min to remove the OCT compound, washed with 50% (v/v) ethanol for 30 sec, and then stained with 2% (w/v) Sudan IV for 1 hr. After further washing with 50% (v/v) ethanol and pure water for 2 min, the slices were counterstained with hematoxylin. Photographs were acquired using a microscope equipped with a 10-fold magnification objective and quantified on an Alpha Imager 2200 documentation system (Alpha Innotech, USA). The manifestation of fatty liver progression was presented as the percentage of the area of oil droplets to the total liver tissues (cells).

### 2.6. Aortic Fatty Streak Staining

The aortas were opened longitudinally to expose the intimal surface and rinsed gently with normal saline. Aortas were incubated in 2% (w/v) Sudan IV, rinsed with several concentrations (100%, 90%, 80%, 70%, and 60%) of ethanol for 1 min, and then rinsed with pure water. The photographs were acquired using a digital camera (Nikon D80, Japan) and quantified on an Alpha Imager 2200 documentation system (Alpha Innotech, USA). The progression of the fatty streak lesions was presented as the percentage of the stained area to the total area.

### 2.7. Western Blot

Proteins extracted from the frozen liver tissues were subjected to SDS-PAGE under reducing conditions on 10% acrylamide gels and transferred to polyvinylidene fluoride (PVDF) membranes by electroblotting. After blockade of nonspecific binding sites, membranes were incubated with primary antibodies (1 : 1,000 dilution), followed by horseradish peroxidase-conjugated secondary antibodies (1 : 2,000 dilution). Protein expression was visualized using SuperSignal West Pico Chemiluminescent Substrate (Thermo Scientific, USA), and the luminescence signal was acquired and analyzed with a Fujifilm LAS-4000 system (Japan). The amounts of p-AMPK*α*, p-ACC, and HMGCR were expressed relative to the amount of *β*-actin (loading control).

### 2.8. Statistical Analysis

All values are expressed as mean ± standard deviation (SD). Data were compared with a one-way analysis of variance (ANOVA) to evaluate differences among multiple groups. A value of *P* < 0.05 was considered statistically significant.

## 3. Results

### 3.1. Regulatory Effect of VT Extract on Serum Lipids

**Figure 1:**
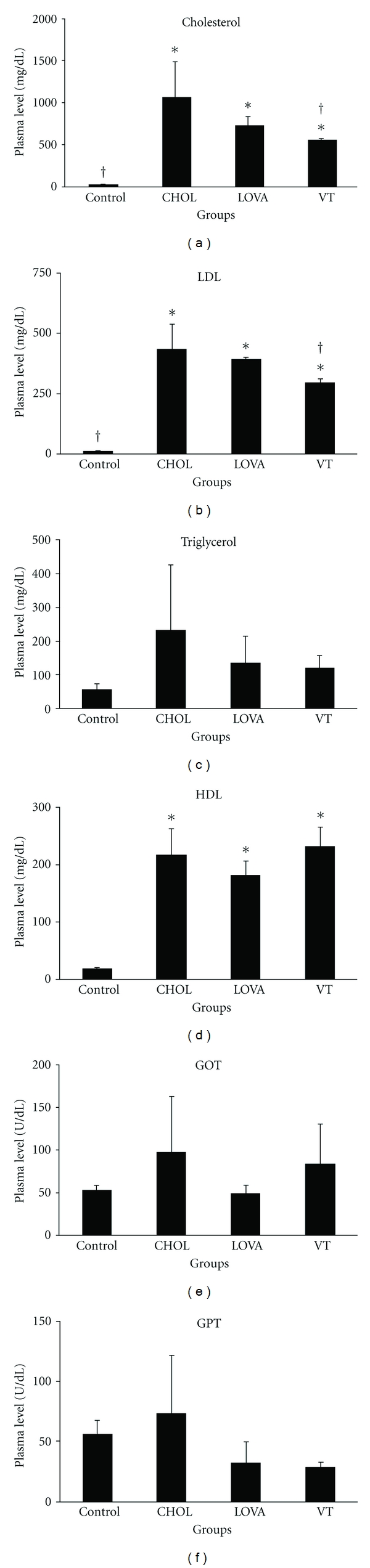
Blood chemistry parameters were measured in the hypercholesterolemic rabbit model after the 8-week study. Control group: regular diet; CHOL group: 0.5% (w/w) cholesterol diet alone; LOVA group: 0.5% (w/w) cholesterol diet with 0.01% (w/w) lovastatin; VT group: 0.5% (w/w) cholesterol diet with 7% (w/w) *V. thunbergii* extract. * and ^†^ indicate a *P* < 0.05 as compared with the control group and CHOL group, respectively.

The blood chemistry parameters were examined to evaluate whether the VT extract could reduce serum lipids (Figure 1). Our data revealed that the plasma level of cholesterol, LDL, triglycerides, HDL, GOT, and GPT did not significantly vary among the different groups prior to study (data not shown). At the end of the 8-week study, our results showed that a 0.5% (w/w) cholesterol diet markedly stimulated plasma levels of cholesterol, LDL, and HDL (Figures 1(a), 1(b), and 1(d)) and slightly increased triglyceride levels without statistical significance (Figure 1(c)). Under the same conditions, a 7% (w/w) VT extract significantly reversed a 0.5% (w/w) cholesterol diet-induced accumulation of serum lipids, which was similar to the effect in the LOVA group (Figures 1(a) and 1(b)). Besides, the serum LDL/HDL ratio was increased by 0.5% (w/w) cholesterol diet (ratio = 1.97) as compared with control group (ratio = 0.36), which can be reversed by 7% (v/v) VT supplement (ratio = 1.26). Furthermore, the experimental data also demonstrated that a 7% (w/w) VT extract supplement did not cause obvious liver damage or toxicity, which was determined by examining plasma levels of GOT and GPT at the end of the 8-week study (Figures 1(e) and 1(f)).

### 3.2. Regulatory Effect of VT Extract on Fatty Liver Disease and Lipid Accumulation

**Figure 2:**
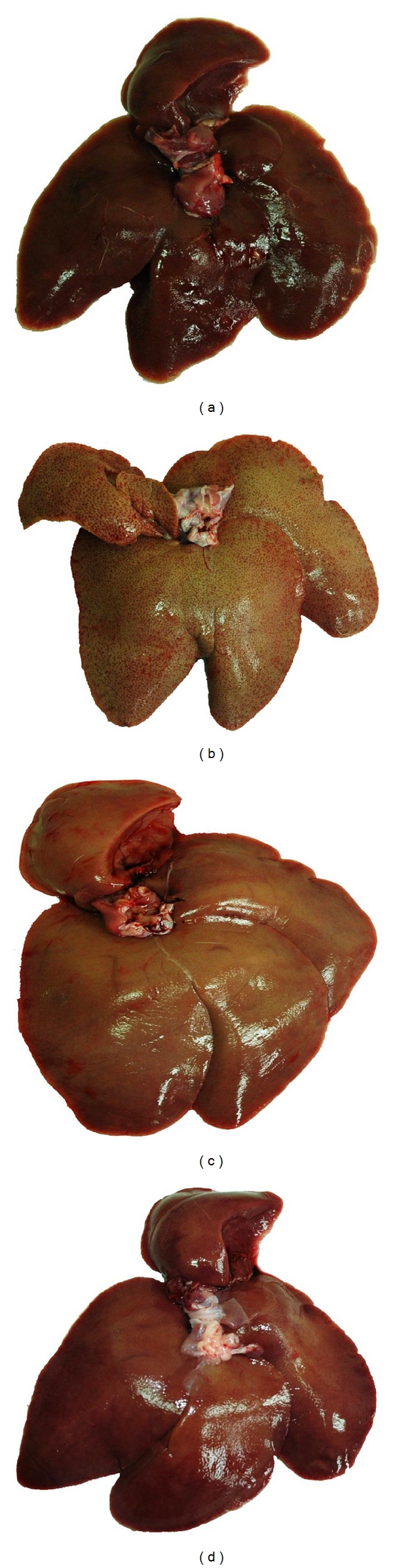
Photographs of liver appearance in the hypercholesterolemic rabbit model after the 8-week study. (a) Control group; (b) CHOL group; (c) LOVA group; (d) VT group.

**Figure 3:**
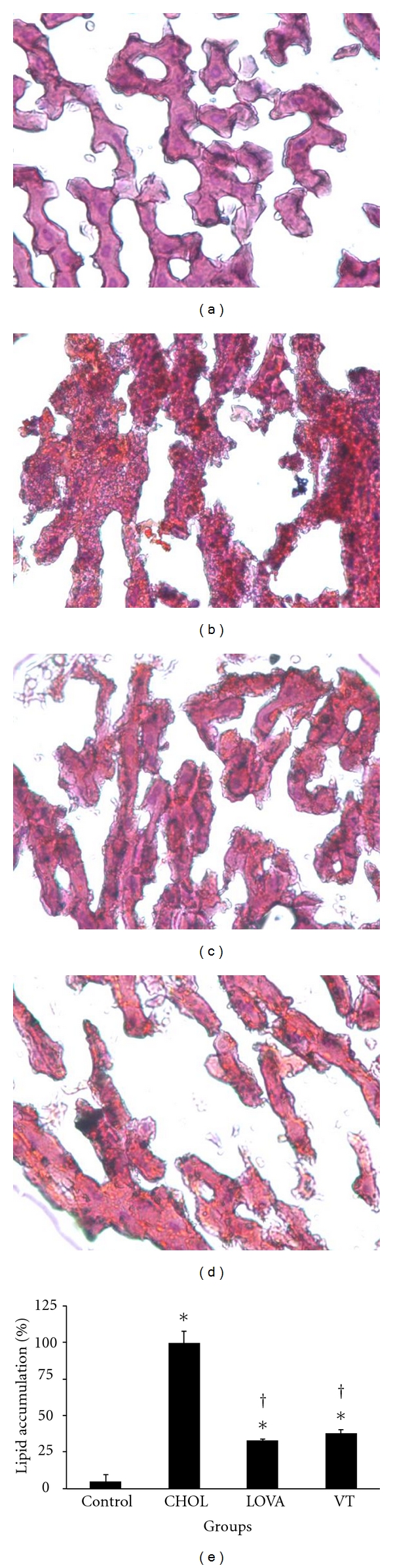
Histopathochemical examination of liver tissues in the hypercholesterolemic rabbit model after the 8-week study. (a) Control group; (b) CHOL group; (c) LOVA group; (d) VT group; (e) relative lipid accumulation within liver tissues among different groups. * and ^†^ indicate a *P* < 0.05 as compared with the control group and CHOL group, respectively.

After the 8-week study, a histopathological analysis of liver cryosections was performed in order to determine whether the VT extract could prevent the formation of fatty liver (Figure 2) and lipid accumulation within liver tissues (Figure 3). Our data demonstrated that a 0.5% (w/w) cholesterol diet could induce phenomena resembling fatty liver (Figure 2(b)) and a 7% (w/w) VT extract reduced the severity of cholesterol diet-induced fatty liver (Figure 2(d)). Similarly, a 0.5% (w/w) cholesterol diet also increased lipid (or oil droplets) accumulation within the liver tissues (Figures 3(b) and 3(e)), and this effect was markedly reversed by a 7% (w/w) VT extract supplement (Figures 3(d) and 3(e)), which was similar to the results of the LOVA group (Figures 3(c) and 3(e)).

### 3.3. Regulatory Effect of VT Extract on Atherosclerosis Formation

**Figure 4:**
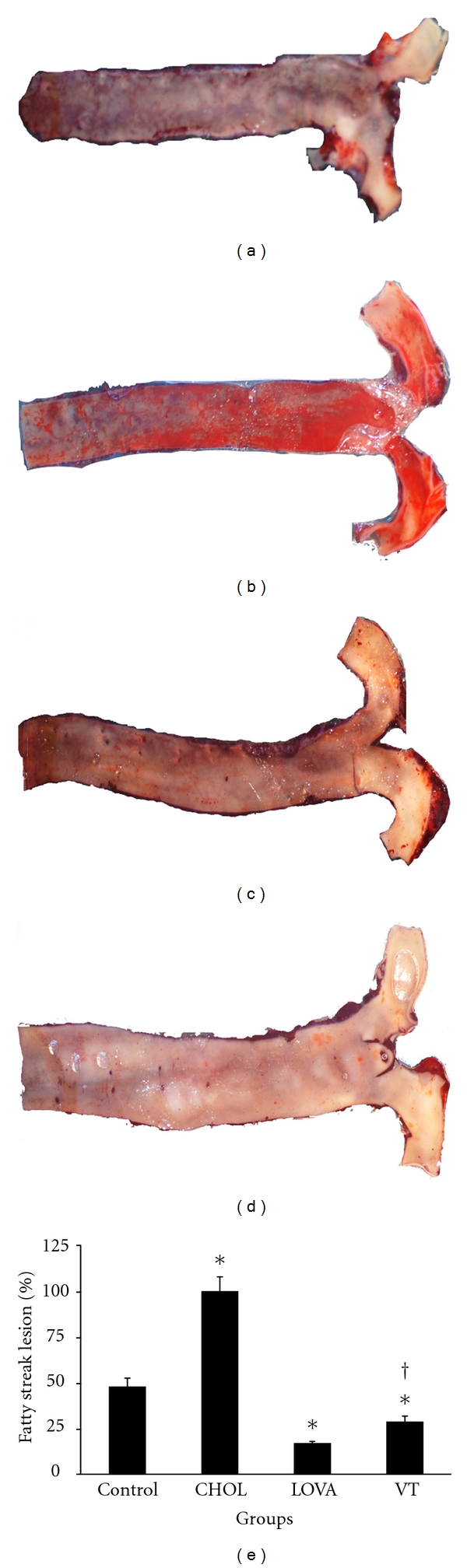
Histopathochemical examination of aortic fatty streak lesions in the hypercholesterolemic rabbit model after the 8-week study. (a) Control group; (b) CHOL group; (c) LOVA group; (d) VT group; (e) relative area of fatty streak lesion on aortic intima among different groups. * and ^†^ indicate a *P* < 0.05 as compared with the control group and CHOL group, respectively.

Sudan IV staining of the fatty streak lesions within the aorta was used to estimate whether the VT extract could reduce the formation of atherosclerosis plaques after the 8-week study (Figure 4). The present study showed that a 0.5% (w/w) cholesterol diet could dramatically increase aortic fatty streak lesions as compared with the control group (Figures 4(a), 4(b), and 4(e)). In addition, a 7% (w/w) VT extract markedly reduced the intensity of fatty streaks on the aorta intima as compared with the CHOL group (Figures 4(b), 4(d), and 4(e)), which was the same result as the LOVA group (Figures 4(b), 4(c), and 4(e)). 

### 3.4. Regulatory Effect of VT Extract on Lipid Metabolism-Associated Proteins

**Figure 5:**
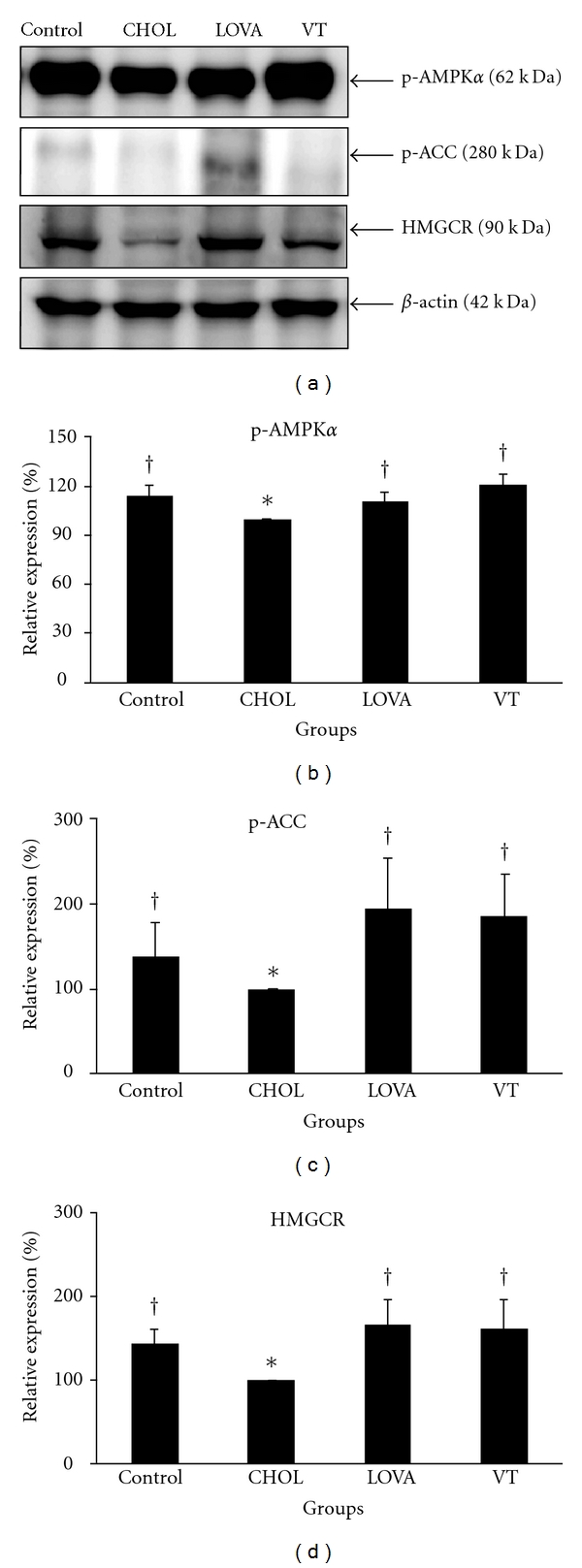
Protein expression of lipid metabolism associated molecules in the hypercholesterolemic rabbit model after the 8-week study. * and ^†^ indicate a *P* < 0.05 as compared with the control group and CHOL group, respectively.

Lipid metabolism-associated proteins such as AMPK, ACC, and HMGCR were examined to determine the molecular mechanisms underlying these VT extract-induced lipid-lowering effects (Figure 5(a)). Experimental data revealed that the 8-week treatment with the 0.5% (w/w) cholesterol diet significantly decreased the protein expression of phospho-AMPK (Figure 5(b)), phospho-ACC (Figure 5(c)), and HMGCR (Figure 5(d)). The addition of a 7% (w/w) VT extract clearly returned the expression of these proteins to near basal levels as compared with the CHOL group, which was the same result as the LOVA group.

## 4. Discussion

Cardiovascular diseases such as coronary heart disease and myocardial infarction are complications of atherosclerosis. In addition, abnormalities in lipid metabolism and coagulation are major contributors to the pathology of atherosclerosis. *V. thunbergii* has been demonstrated to provide many cardiac benefits that may be modulated through some of its identified components, which include the following compounds: *β*-sitosterol, *β*-sitosterol-3-O-*β*-D-glucoside, ampelopsin C, betulinic acid, botulin, caffeic acid, friedelin, heyneanol A, l-dotriacontanol, lupeol, luteolin-7-O-glucoside, miyabenol A, narcissin, oligostilbenes, piceatannol, quercetin-3-O-galactoside, quercitrin, rutin, stigmasterol, triacontanoic acid, vanillic acid, viniferin, viniferal, vitisin A, vitisin C, and vitisinols A-D [11–13, 16, 17]. Of these components, ampelopsin C, miyabenol A, viniferin, viniferal, vitisin A, vitisin C, and vitisinols A-D belong to the class of resveratrol derivatives. Resveratrol is a polyphenolic antioxidant that is found in grape skin and is strongly related to the cardioprotective effect of red wine [18], which is a potent atheroprotective compound [19, 20]. Growing evidences suggest that resveratrol protects the cardiovascular system in numerous ways, including antiapoptotic [21, 22], antiplatelet [11, 23, 24], antioxidative [25–27], and antiinflammatory effects [28–30], as well as regulating lipid metabolism [20, 31].

The regulation of lipid metabolism by resveratrol is related to the modulation of lipid turnover, inhibition of eicosanoid production, and prevention of low-density lipoprotein oxidation [31]. Several *in vivo* studies have revealed that resveratrol can reduce plasma levels of LDL, cholesterol, and triglycerides in high cholesterol diet-induced hypercholesterolemia in rodent models [32–34]. Similarly, long-term resveratrol administration can also decrease plasma levels of triglycerides, total cholesterol, and free fatty acids and hepatic total lipids in obese Zucker rats [35]. Ahn et al. [36] demonstrated that a supplementary diet with resveratrol can reduce atherosclerotic lesions and found that it decreased the levels of total hepatic lipids and triglycerides as well as their accumulation. In addition, mRNA expression and enzymatic activity of hepatic HMG-CoA reductase can also be downregulated by treatment with resveratrol, which may result in decreased cholesterol synthesis [37, 38]. *In vitro* studies have also shown promising beneficial effects of resveratrol on lipid metabolism. In isolated normal rat hepatocytes, resveratrol reduced the synthesis of fatty acids and triglycerides, which suggests a possible mechanism underlying resveratrol's effects in reducing the levels of triglycerides and other lipoprotein in the circulation [39]. Goldberg et al. [40] indicated that resveratrol effectively reduced LDL production and modulated hepatic lipid metabolism via decreasing secretion of triglycerides and cholesterol esters in HepG2 cells. Our results indicated that the VT extract reduced the plasma levels of cholesterol and LDL (Figure 1), decreased lipid accumulation in liver tissues (Figures 2 and 3), and diminished aortic fatty streak lesions (Figure 4). These lipid-lowering effects of the VT extract may be partially explained by the activities of the resveratrol derivatives mentioned previously.

Epidemiological studies have consistently shown that serum HDL concentration is inversely correlated with the incidence of cardiovascular disease. However, it appears that the relationship between HDL and CVD risk is more complex and not just merely related to the serum HDL levels. Barter et al. [41] noted that torcetrapib can markedly increase the serum HDL, but the results of clinical trial showed to increase the risk of deaths and cardiac events in patients receiving torcetrapib. Several studies also showed that serum HDL levels are not always consistent with the incidence of CVD [42, 43]. In the present study, the results showed that high-cholesterol diet can markedly induce the serum levels of both LDL and HDL (Figures 1(b) and 1(d)), whose effects have be reported by others [44, 45]. Thereby, evaluating CVD risk on the basis of serum HDL concentration alone may be misleading, and the serum LDL/HDL ratio should also be evaluated together for CVD risk assessment. Our data showed that the high-cholesterol diet leads to a five-fold increase in the serum LDL/HDL ratio as compared with control group and VT supplement can reverse this parameter to support its lipid-lowering effect in the present study.

AMPK plays a critical role in the modulation of lipid metabolism by inhibiting the activation of ACC and HMGCR, which results in the acceleration of fatty acid oxidation and the suppression of cholesterol biosynthesis. Numerous studies have indicated that the stimulating activity of the AMPK pathway can effectively regulate lipid metabolism. Adiponectin is an adipocyte-derived adipokines and can protect against alcoholic fatty liver disease via the activation of the SIRT1- (sirtuin 1-) AMPK pathway [46]. Kusakabe et al. reported that leptin decreased liver and skeletal muscle triacylglycerol content, which was accompanied by an increase of AMPK activity in skeletal muscle [47]. Lin et al. found that theaflavins significantly reduced lipid accumulation, suppressed fatty acid synthesis, and stimulated fatty acid oxidation via stimulating AMPK and then inhibiting ACC activity [48]. Luteolin is another component identified from *V. thunbergii* that has been shown to reduce lipid accumulation in HepG2 cells. This effect may be partially mediated by that activation of the AMPK signaling pathway, which upregulates carnitine palmitoyl transferase 1 (CPT-1) and downregulates sterol regulatory element binding protein 1c (SREBP-1c) and FAS gene expression [49]. Metformin is an oral antidiabetic drug that has been demonstrated to lower the hepatic lipid content by activating AMPK, which may mediate the beneficial effects of this drug in hyperglycemia and insulin resistance [50, 51]. In addition, a number of studies have also demonstrated that resveratrol treatment can stimulate AMPK activity by regulating lipid metabolism [35, 52–54]. In the present study, the data showed that a VT extract could stimulate phosphorylation of AMPK and ACC (Figure 5). However, this effect may be partially mediated by some of the bioactive components of the VT extract, such as luteolin and the resveratrol derivatives mentioned previously. On the other hand, previous studies have demonstrated that activated AMPK will phosphorylate and then inactivate HMGCR [7]. Nevertheless, the total protein expression of HMGCR was upregulated by supplementation with the VT extract in the present study. HMGCR is regulated via a negative feedback mechanism that is mediated by sterols and nonsterol metabolites derived from mevalonate. Moreover, this enzyme is suppressed by cholesterol in mammalian cells [55–57]. Therefore, the upregulation of HMGCR protein after an 8-week dietary supplement with the VT extract may have been triggered by the low levels of serum cholesterol (Figure 1).

## 5. Conclusions

Our experimental study revealed that a VT extract supplement can reduce serum LDL/HDL ratio as well as plasma levels of cholesterol and LDL, decrease lipid accumulation in tissues, and diminish aortic fatty streak lesions. Moreover, the lipid-lowering effects of the VT extract may have been partially mediated by the activation of AMPK, which was followed by the inhibition of the activation of ACC. These results suggest that *V. thunbergii* may have the potential to be developed as a lipid-lowering therapeutic agent or medicinal health food for the prevention or treatment of cardiovascular diseases such as atherosclerosis.
